# Outbreak of *Kodamaea ohmeri* fungemia in neonates: case series and literature review

**DOI:** 10.3389/fmicb.2026.1810513

**Published:** 2026-04-07

**Authors:** Kehong Lou, Xinzhu Zhou, Yiyun Xu, Yanbing Li, Wei Kang, Ge Zhang, Jin Li, Tong Wang, Haotian Gao, Xiangyang Chen, Yingchun Xu, Meng Xiao, Menglan Zhou

**Affiliations:** 1 State Key Laboratory of Complex Severe and Rare Diseases, Department of Clinical Laboratory, Peking Union Medical College Hospital, Peking Union Medical College, Chinese Academy of Medical Sciences, Beijing, China; 2 Department of Clinical Laboratory, Ningbo No.2 Hospital, Ningbo, Zhejiang, China; 3 Beijing Key Laboratory for Mechanisms Research and Precision Diagnosis of Invasive Fungal Diseases, Beijing, China; 4 Graduate School, Peking Union Medical College, Chinese Academy of Medical Sciences, Beijing, China; 5 Zhengzhou People’s Hospital, The Fifth Clinical Medical College of Henan University of Chinese Medicine, Zhengzhou, China

**Keywords:** antifungal susceptibility, fungemia, invasive fungal infections, *Kodamaea ohmeri*, microsatellite genotyping, neonates, nosocomial infection

## Abstract

**Introduction:**

*Kodamaea ohmeri* is an emerging opportunistic yeast pathogen associated with high mortality yet reports of nosocomial outbreaks—particularly in neonatal intensive care units—remain scarce.

**Methods:**

We retrospectively identified five cases of *K. ohmeri* fungemia occurring within a 2-month period in the NICU of a tertiary general hospital in China. Clinical and epidemiological data were collected. Isolates were identified by MALDI-TOF MS and characterized by CHROMagar™ Candida chromogenic medium, antifungal susceptibility testing (Sensititre YeastOne™), and microsatellite genotyping. A systematic literature review of *K. ohmeri* bloodstream infections was also performed.

**Results:**

All five neonates (median age 1 month) had indwelling catheters, received mechanical ventilation and broad-spectrum antibiotics, and all survived following antifungal therapy and catheter removal. Seven isolates from the five patients exhibited identical microsatellite genotypes across three highly polymorphic loci, confirming clonal transmission and a common nosocomial origin. Morphological dimorphism (smooth/rough colony types) was observed in one isolate. Fluconazole MICs were elevated (4–8 μg/mL), whereas echinocandins and amphotericin B showed low MICs. The literature review identified 30 sporadic cases and two prior outbreaks (total 38 patients; mortality 32%), with prematurity, indwelling catheters, and broad-spectrum antibiotic use as predominant risk factors.

**Conclusion:**

This cluster describes an outbreak of *K. ohmeri* fungemia in neonates. Microsatellite genotyping proved valuable for confirming nosocomial transmission. Accurate identification (MALDI-TOF MS), prompt catheter removal, and appropriate antifungal therapy are crucial. Enhanced hand hygiene and environmental disinfection remain essential for NICU infection control.

## 1 Introduction

Invasive fungal infections (IFIs), characterized by high morbidity and mortality, are systemic infections caused by various opportunistic fungi, including yeasts (e.g., *Candida* spp.), molds (e.g., *Aspergillus* spp., *Mucorales*), and diphasic fungi (e.g., *histoplasma* spp.) (Fang et al., 2023). *Candida* species account for approximately 70% of IFI cases (Bongomin et al., 2017). Although *Candida* remains the most prevalent pathogen, the incidence of infections caused by rare, non-*Candida* opportunistic yeast species is increasing (Mpakosi et al., 2024). *Kodamaea ohmeri* (*K. ohmeri*) is one of the most significant emerging pathogens among these rare yeasts, associated with higher mortality rates compared to candidemia (Mpakosi et al., 2024).

*K. ohmeri*, also known as *Pichia ohmeri* or *Yamadazyma ohmeri*, belongs to the *Saccharomycetaceae* family. It is frequently isolated from environmental sources and is predominantly utilized in the food fermentation industry (Zhou et al., 2021). *K. ohmeri* was firstly isolated from pleural effusion in 1984 and initially considered a contaminant. Since its first report as a cause of fungemia in the United States in 1998 (Bergman et al., 1998), *K. ohmeri* has been recognized as an emerging human pathogen capable of causing infections in immunocompromised patients, neonates, and long-term hospitalized individuals (Sun et al., 2024; Vivas et al., 2016). Currently, sporadic cases of *K. ohmeri* infection have been reported worldwide, but reports of cluster infections remain scarce.

In the present study, our team retrospectively identified five cases of *K. ohmeri* fungemia occurring within a 2-month period in the NICU of a municipal tertiary general hospital in China. Microsatellite genotyping confirmed clonal transmission, indicating a nosocomial outbreak. We further summarized the clinical, epidemiological, and microbiological characteristics of this outbreak and conducted a literature review, aiming to contribute to the prevention and management of invasive fungal diseases caused by *K. ohmeri*.

## 2 Materials and methods

### 2.1 Isolate selection

The seven *K. ohmeri* isolates from five neonates in this outbreak were originally collected through the CHIF-NET program and included in a previous study by Li et al. (2024), where a potential outbreak was first identified based on microsatellite genotyping results. The present study provides a detailed clinical and microbiological analysis of that outbreak. Inclusion criteria for this case series were: (1) isolation of *K. ohmeri* from blood culture; (2) admission to the NICU during the outbreak period (June–August 2017); and (3) availability of clinical records and isolates for further analysis.

### 2.2 Clinical data collection

A retrospective collection of epidemiological, clinical, and microbiological data was conducted for five confirmed cases of *K. ohmeri* fungemia occurring between June and August 2017 in the neonatal intensive care unit (NICU) of a municipal tertiary general hospital in China.

Epidemiological data primarily included patient demographic characteristics: age, sex, and date of admission. Clinical data encompassed: (1) underlying diseases; (2) clinical manifestations; (3) risk factors for invasive fungal infection; (4) clinical management; and (5) clinical outcomes. Microbiological data included: (1) source and time of pathogen isolation; and (2) antifungal treatment regimens.

All data were reviewed and validated by two independent investigators and were collected in accordance with the ethical standards of the institutional research committee.

### 2.3 Isolation and identification

Strains isolated from distinct sites of the same patient were classified as different strains. A total of 7 strains were isolated from the 5 patients (Patients 1 and 5 both had pathogens isolated simultaneously from peripheral and catheter blood cultures).

All isolates were inoculated onto CHROMagar™ Candida chromogenic medium (CHROMagar, France) and incubated at 35 and 28°C in ambient air. Colony morphology and color changes were observed at 24, 48, and 72 h and at 7 days. Gram staining and calcofluor white fluorescence staining were performed for microscopic examination.

Definitive identification was performed using matrix-assisted laser desorption/ionization time-of-flight mass spectrometry (MALDI-TOF MS) with the VITEK MS IVD instrument (BioMerieux, France) according to the manufacturer’s protocol. Analysis utilized the VITEK^®^ MS IVD v2.0 database.

### 2.4 Microsatellite genotyping

Yeast genomic DNA was extracted from colonies using the MasterPure™ Yeast DNA Purification Kit (LGC, United Kingdom). Based on the K. ohmeri reference strain NRRL Y-1932 (GenBank assembly accession: GCA_003708155.1 ASM370815v1) genome, 842 microsatellite loci were identified. Primers for 50 loci were designed, and three highly polymorphic loci (P10, P11, P26) located in non-coding regions were selected following reliable amplification and Polyacrylamide Gel Electrophoresis (PAGE) assessment in pilot experiments. The detailed development and validation of these markers have been described in our previous work (Li et al., 2024)

All strains underwent Polymerase Chain Reaction (PCR) amplification, and allele sizes were precisely determined using an ABI 3730XL sequencer. Genetic relatedness was analyzed using BioNumerics software v7.6 (Applied Maths, Sint-Martens-Latem, Belgium).

### 2.5 Antifungal susceptibility testing

*In vitro* antifungal susceptibility of the seven *K. ohmeri* strains was determined using the Sensititre YeastOne™ YO10 panel (Thermo Scientific, Cleveland, OH, United States). Minimum inhibitory concentrations (MICs) were determined for anidulafungin, micafungin, caspofungin, flucytosine, posaconazole, voriconazole, itraconazole, fluconazole, and amphotericin B. As neither the European Committee on Antimicrobial Susceptibility Testing (EUCAST) nor the Clinical and Laboratory Standards Institute (CLSI) provide specific breakpoints for K. ohmeri, results are presented as MIC values without interpretive categories.

### 2.6 Literature review

A systematic literature search was conducted in PubMed using the keywords: “(*Kodamaea ohmeri*” OR “*Pichia ohmeri*”) AND (“bloodstream infection” OR “sepsis” OR “bacteremia” OR “fungemia” OR “septicopyemia” OR “septicemia”).” Inclusion criteria: (1) original case reports or case series; (2) documented *K. ohmeri* bloodstream infection; (3) published in English or Chinese; (4) contained complete clinical details. Exclusion criteria: reviews, conference abstracts, articles lacking complete clinical information, or not describing bloodstream infection.

## 3 Results

### 3.1 Epidemiological description of the outbreak

**FIGURE 1:**
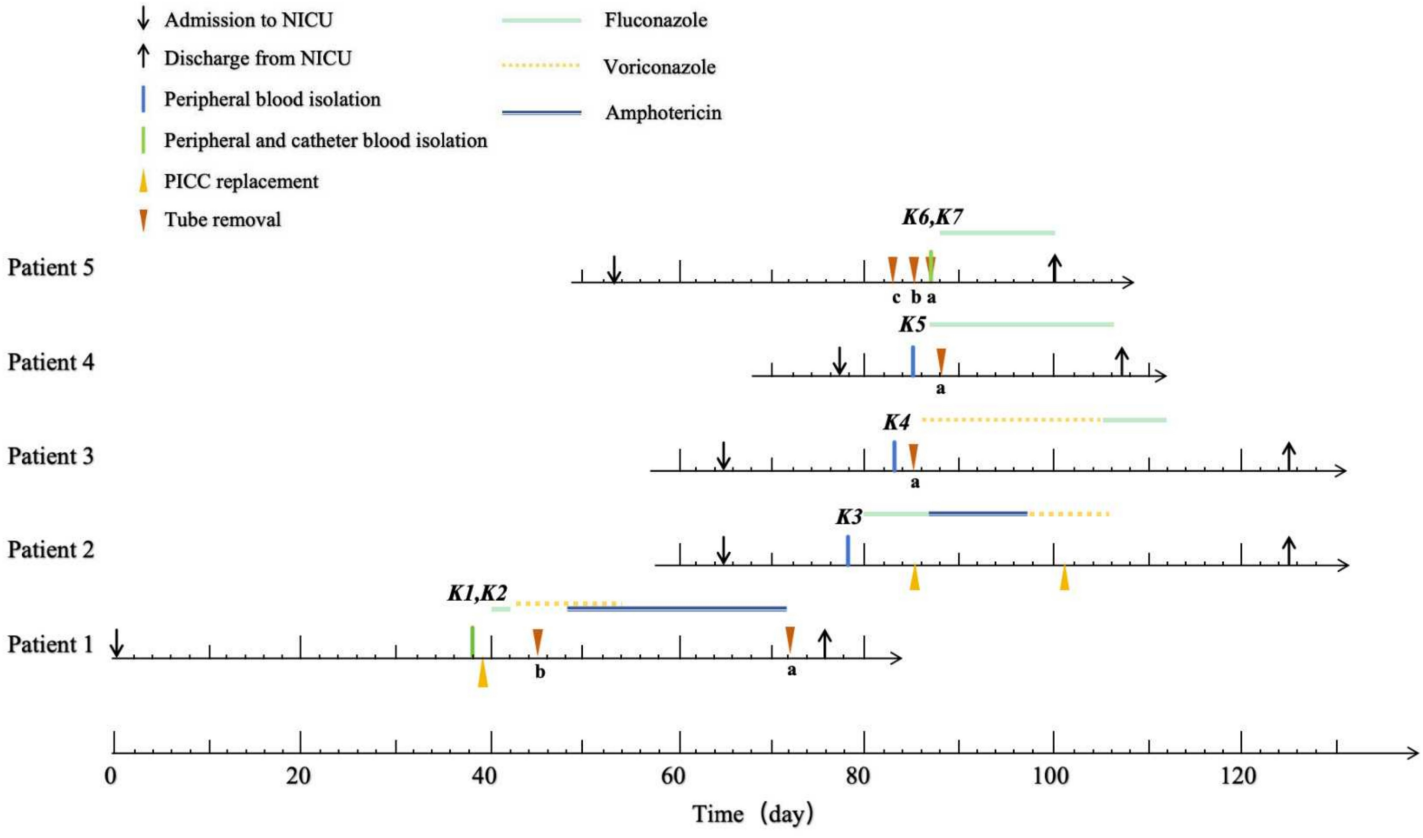
Timeline of the isolation and the antifungal treatments of *K. ohmeri* in five patients. *^a^*PICC removal. *^b^*Abdominal drain removal. *^c^*Gastric tube removal.

Five patients were admitted to the NICU; two were males and three were females. All were under 1 year of age, with a mean age of 3 months. Patient 1, who had undergone colostomy for congenital anal atresia, was the index case, admitted to the NICU on June 13, 2017. Pathogens were isolated from both peripheral and catheter blood culture on hospital day 38. Patient 2 was admitted on August 17, 2017, for severe community-acquired pneumonia and neonatal sepsis. *K. ohmeri* was detected in positive peripheral blood culture on hospital day 13. Patient 3 was admitted on the same day as Patient 2 due to severe congenital duodenal stenosis with obstruction. The same pathogen was isolated from peripheral blood culture 5 days after its detection in Patient 2. Patients 4 and 5 were admitted for congenital anal agenesis and congenital duodenal atresia, respectively. Pathogens were detected in Patient 4’s peripheral blood culture 2 days after detection in Patient 3, and in both the peripheral and catheter blood culture in Patient 5 4 days after Patient 3. Final identification confirmed *K. ohmeri* infection in all five patients. Figure 1 presents the timeline of the isolation and antifungal treatment of *K. ohmeri* strains for the five patients.

### 3.2 Clinical characteristics and management

Primary clinical manifestations included cough, fever, vomiting, and diarrhea. Patient 2 exhibited dyspnea, while Patient 3 presented with anemia and progressive thrombocytopenia. All patients received mechanical ventilation and corticosteroid therapy. In addition to peripherally inserted central catheters (PICCs), four patients were placed gastric tubes and two were placed abdominal drainage tubes. All patients received empiric therapy with multiple antibiotics (≥ 2). Cefoperazone-sulbactam and teicoplanin were the most frequently administered antibiotics (5/5 patients), followed by piperacillin-tazobactam (4/5), imipenem (4/5), azithromycin (2/5), and cefoxitin (2/5). Patient 4 additionally received cefepime. PICCs were removed or replaced within 2 days of pathogen detection in all cases. Antifungal therapy consisted of fluconazole for all five patients, with three also receiving voriconazole and two receiving amphotericin B. Following active treatment, all patients were discharged after a mean hospital stay of 54.8 days. Table 1 lists the clinical and epidemiological characteristics of five patients in this study.

**TABLE 1:** Clinical and epidemiological characteristics of the *K. ohmeri* fungemia patient in present case.

Clinical characteristics	Patient 1	Patient 2	Patient 3	Patient 4	Patient 5
Age (months)	12	2	1	1	1
Gender	Male	Female	Female	Male	Male
Admission time	2017.6.13	2017.8.17	2017.8.17	2017.8.29	2017.8.5
Department	NICU	NICU	NICU	NICU	NICU
Underlying disease	CHD, CIA, bronchitis	Severe CAP, sepsis shock	CDSO, bronchopneumonia	CIA, ileus, bronchopneumonia	CDA, bronchopneumonia
Clinical manifestation	Cough	Fever, wheezing, diarrhea, dyspnea	Vomiting, cough	Abdominal distension, fever	Vomiting, cough
Hospital stays (days)	76	60	60	31	47
Isolation sources	Peripheral blood (K1), catheter blood (K2)	Peripheral blood (K3)	Peripheral blood (K4)	Peripheral blood (K5)	Peripheral blood (K6), catheter blood (K7)
Isolation time	2017.7.21	2017.8.30	2017.9.4	2017.9.6	2017.9.8
Antifungal agents	Fluconazole, Voriconazole, Amphotericin B	Fluconazole, Amphotericin B, Voriconazole	Voriconazole, Fluconazole	Fluconazole	Fluconazole
Antibiotic	TZP, CSL, TEC, IPM, AZM, FOX	CSL, TEC, IPM, AZM, TZP, LZD, MEM	TZP, CSL, TEC, IPM, FOX	FEP, CSL, TEC	TZP, ERY, IPM, TEC, CSL
Catheter	PICC, AD	PICC, OGT	PICC, OGT	PICC, OGT	PICC, AD, OGT
Tube removal	Yes	Yes	Yes	Yes	Yes
Mechanical ventilation	Yes	Yes	Yes	Yes	Yes
Corticosteroids	Budesonide, dexamethasone	Budesonide	Budesonide	Budesonide	Budesonide
Outcome	Recovered	Recovered	Recovered	Recovered	Recovered

AD, Abdominal Drain; CHD, Congenital Heart Disease; CIA, Congenital Imperforate Anus; CAP, Community-Acquired Pneumonia; CDS, Congenital Duodenal Stenosis; CDSO, Congenital Duodenal Stenosis with Obstruction; CDA, Congenital Duodenal Atresia; OGT, Orogastric Tube; TZP, Piperacillin-tazobactam; CSL, Cefoperazone/Sulbactam; TEC, Teicoplanin; IPM, Imipenem; AZM, Azithromycin; FOX, Cefoxitin; LZD, Linezolid; MEM, Meropenem; ERY, Erythromycin.

### 3.3 Microbiological characteristics

**FIGURE 2:**
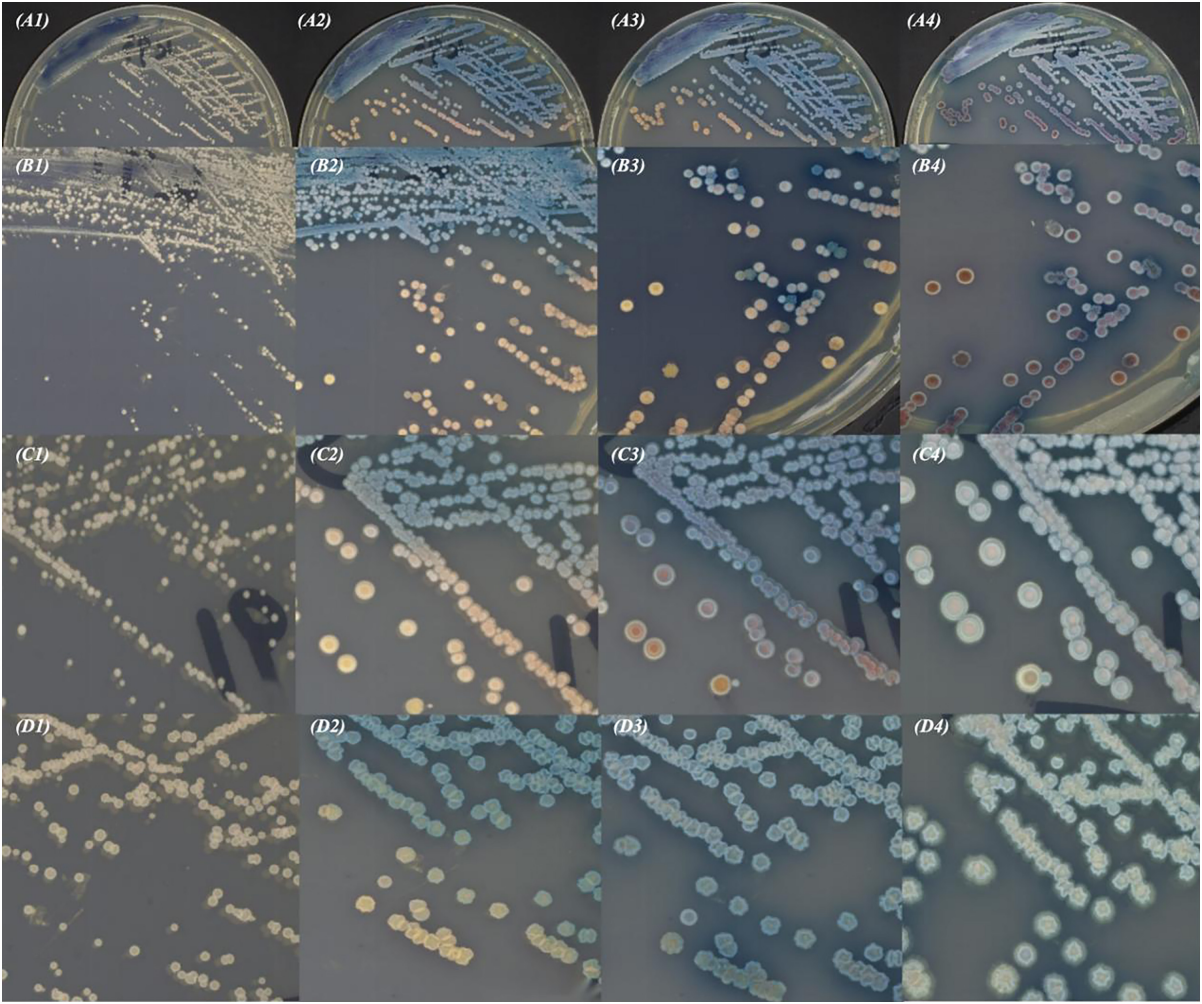
Morphology of *K. ohmeri* strains incubated on CHROMagar Candida chromogenic medium- strain *K1* **(A)**, strain *K5* **(B)**, smooth type of *K5* **(C)**, rough type of *K5* **(D)** after (1) 24 h, (2) 48 h, (3) 72 h, and (4) 7 days.

**FIGURE 3:**
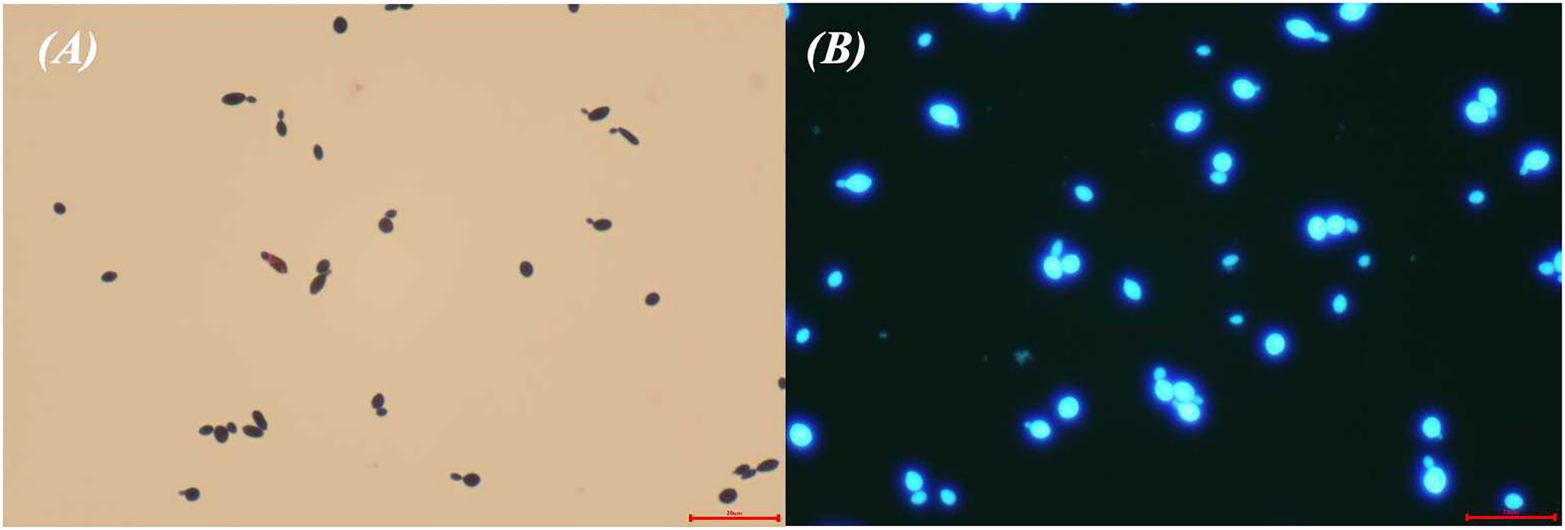
Gram staining **(A)** (× 40) and calcofluor white fluorescence staining **(B)** (× 40) of *K. ohmeri*.

On CHROMagar™ Candida chromogenic medium, colonies appeared pink at 24 h, turned blue-purple at 48 h, and became blue at 72 h (Figure 2). Gram staining and calcofluor white fluorescence staining revealed yeast-like cells (Figure 3).

**FIGURE 4:**
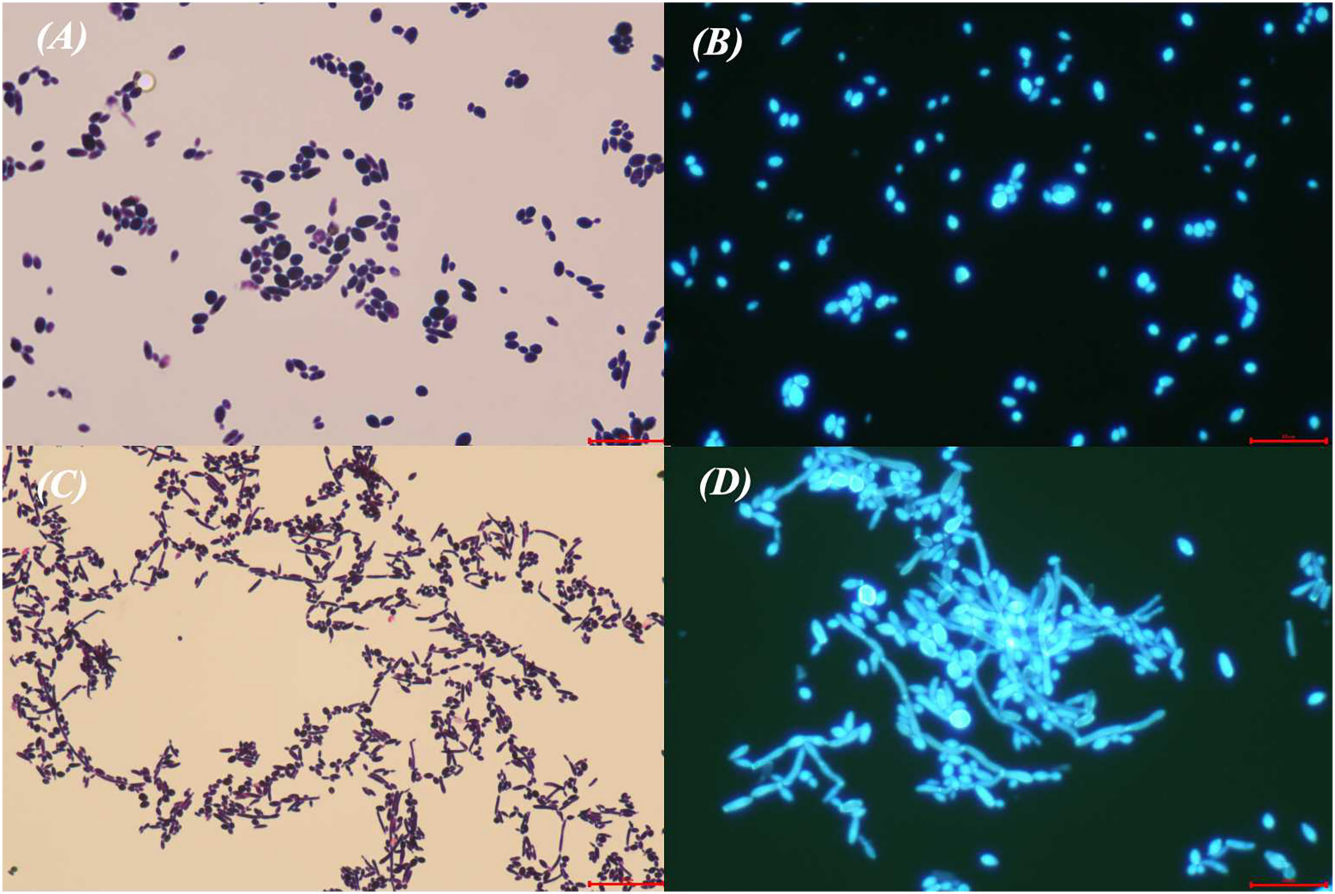
Microscopic examination of *K. ohmeri (K5)*. Smooth type: **(A)** Gram staining, (× 40); **(B)** Calcofluor white staining (× 40). Rough type: **(C)** Gram stain (× 10); **(D)** Calcofluor white staining (× 40).

Notably, the *K. ohmeri* K5 strain isolated from peripheral blood of Patient 4 exhibited two distinct colonial morphologies: a smooth type (S) with a flat, smooth surface, and a rough type (R) with a rough and wrinkled surface. Microscopic examination of the S-type revealed yeast-like cells, while the R-type revealed predominantly pseudohyphae (Figure 4).

MALDI-TOF MS analysis using the VITEK^®^ MS IVD v2.0 database definitively identified all isolates as *K. ohmeri*. Initial presumptive identification based on CHROMagar color had misidentified six isolates as *C. albicans* and one isolate as *C. dubliniensis*.

### 3.4 Microsatellite genotyping

All seven *K. ohmeri* strains isolated from the five patients shared the identical microsatellite genotype across the three highly polymorphic loci (P10, P11, and P26), The specific allele sizes for all strains were 308 base pairs (bp) for locus P10, 367 bp for locus P11, and 311 bp for locus P26. This genotype, designated as microsatellite type 12 (MT12) in our previous multicenter study (Li et al., 2024), provides strong molecular evidence of clonal transmission, confirming a common nosocomial origin for this outbreak.

### 3.5 Antifungal susceptibility profiles

For the echinocandins, MICs of anidulafungin were 0.25 μg/mL (6/7) and 0.5 μg/mL (1/7), and those of micafungin were 0.12 μg/mL (4/7) and 0.25 μg/mL (3/7). Caspofungin MICs ranged from 0.12 to 1 μg/mL, with an MIC50 of 0.25 μg/mL. Among the azoles, posaconazole MICs were 0.12 μg/mL (4/7) and 0.06 μg/mL (3/7), itraconazole MICs were 0.12 μg/mL (3/7) and 0.25 μg/mL (4/7), and voriconazole MIC was uniformly 0.06 μg/mL (7/7). Notably, fluconazole MICs were 8 μg/mL (5/7) and 4 μg/mL (2/7). Both fluconazole MIC values fell within the non-susceptible range according to established *Candida* spp. breakpoints. MICs of 5-fluorocytosine were ≤ 0.06 μg/mL and those of amphotericin B were ≤ 0.25 μg/mL for all strains. The susceptibility profile of all strains is summarized in Table 2.

**TABLE 2:** Antifungal susceptibility results of *Kodamaea ohmeri* isolates.

Antifungal agent	K1	K2	K3	K4	K5	K6	K7
Anidulafungin	0.5	0.25	0.25	0.25	0.25	0.25	0.25
Micafungin	0.25	0.12	0.12	0.12	0.25	0.25	0.12
Caspofungin	0.5	0.25	0.12	0.12	0.25	1	0.12
Flucytosine	< 0.06	<0.06	< 0.06	<0.06	< 0.06	<0.06	< 0.06
Posaconazole	0.12	0.12	0.06	0.06	0.12	0.12	0.06
Voriconazole	0.06	0.06	0.06	0.06	0.06	0.06	0.06
Itraconazole	0.25	0.25	0.12	0.12	0.12	0.25	0.12
Fluconazole	8	8	8	8	4	4	8
Amphotericin B	0.5	0.25	0.25	0.25	0.25	0.25	0.25

### 3.6 Literature review

A systematic literature search identified 54 articles, from which 31 sporadic cases and 3 nosocomial outbreaks of *K. ohmeri* fungemia were included, encompassing a total of 43 patients. The overall mortality rate was 30.2% (13/43). Among these, 15 cases occurred in neonates, with a mortality rate of 20% (3/15), highlighting the vulnerability of this population.

The cohort consisted of 28 males and 15 females. The most frequently reported clinical manifestations were fever and chills (*n* = 35), followed by gastrointestinal symptoms such as vomiting, diarrhea, and abdominal distension (*n* = 11), and respiratory symptoms including cough and dyspnea (*n* = 8). Predominant underlying conditions included prematurity (*n* = 15), pneumonia (*n* = 6), enteritis or necrotizing enterocolitis (*n* = 5), leukemia (*n* = 4), diabetes mellitus (*n* = 4), and severe burns (*n* = 3).

Indwelling catheters were present in 65% (28/43) of patients, and mechanical ventilation was required in 37% (16/43). In 12 patients, *K. ohmeri* was isolated from both blood and catheter tips, supporting a strong association with catheter-related bloodstream infections. Prior exposure to broad-spectrum antibiotics was documented in the majority cases. Two previously reported outbreaks occurred in pediatric intensive care units, and the present study adds a third neonatal outbreak confirmed by molecular genotyping.

These findings indicate that *K. ohmeri* fungemia predominantly affects immunocompromised or critically ill patients, particularly neonates with low birth weight, indwelling devices, and prolonged antibiotic therapy. The high prevalence of catheter-related infections underscores the importance of prompt catheter removal as a key management strategy. Detailed epidemiological and clinical characteristics are summarized in Supplementary Table 1.

## 4 Discussion

*K. ohmeri* fungemia predominantly occurs in patients with severe immunosuppression or immunodeficiency. Common predisposing factors include infectious diseases (e.g., pneumonia, enteritis, bacteremia), hematological malignancies like leukemia, solid tumors, and diabetes. Singh et al. (2024) reported three cases of *K. ohmeri* fungemia in immunocompromised patients with underlying conditions including long-term corticosteroid use, hemodialysis, and extensive burns. Despite prompt initiation of echinocandin therapy, all three patients ultimately died, which was likely attributable to the severity of their underlying conditions and critical illness. Invasive procedures (e.g., central venous catheterization, total parenteral nutrition, mechanical ventilation, PICC insertion) represent significant risk factors. Among the reviewed cases, 28 patients had indwelling catheters (28/43), 16 required mechanical ventilation (16/43), and pathogens were isolated from both catheter tips and blood in 12 patients. Therefore, alongside appropriate antifungal therapy, timely catheter removal or replacement is crucial for managing *K. ohmeri* fungemia. All five patients in this report underwent mechanical ventilation and catheterization, with catheter blood cultures positive in two cases. Prolonged broad-spectrum antibiotic use is another common risk factor, likely due to disruption of normal microbiota and mucosal barrier integrity, facilitating opportunistic yeast invasion. Consequently, discontinuing unnecessary antibiotics upon pathogen identification may constitute an effective adjunctive measure.

Neonates, particularly those with low birth weight or prematurity, are highly susceptible. A review of 342 cases across 39 studies (Mpakosi et al., 2024) indicated that *K. ohmeri* is a leading cause of neonatal fungemia among rare, non-*Candida* opportunistic yeasts (54/342, 16%), with a mortality rate of 39% in affected neonates. Previous reports of neonatal *K. ohmeri* fungemia (Taj-Aldeen et al., 2006; Poojary and Sapre, 2009; Vivas et al., 2016; Sundaram et al., 2011; Al-Sweih et al., 2011) predominantly involved preterm infants, with most weighing < 1,300 g (very low birth weight, VLBW). VLBW preterm infants have immature immune systems, compromised mucocutaneous barriers, prolonged hospitalization, frequent broad-spectrum antibiotic exposure, and undergo multiple invasive procedures, all contributing to *K. ohmeri* fungemia risk. A previous study (Mpakosi et al., 2024) reported that the incidence of neonatal fungemia nearly tripled between 2001 and 2023. The five neonates in our outbreak exhibited all these classic risk factors—including PICC lines, mechanical ventilation, and extensive antibiotic therapy—aligning perfectly with the profiles described in the literature. Concurrently, researchers across multiple regions have verified significantly improved survival rates among very low birth weight (VLBW) and extremely low birth weight (ELBW) infants over the past two decades (AlQurashi, 2021; Abolfotouh et al., 2018; Chen et al., 2014; Santhakumaran et al., 2018). Given the observed epidemiological correlation, investigators hypothesized that the rising incidence of neonatal fungemia may partly reflect improved survival rates of VLBW and ELBW infants, mainly attributable to significant advances in perinatal care. Nevertheless, managing infections in neonatology departments, particularly NICUs, remains challenging.

Preventing nosocomial infections (NIs) is paramount in neonatology, especially within NICUs (Johnson et al., 2021; Baltimore, 1998). The three previously reported in-hospital outbreaks (Otag et al., 2005; Liu et al., 2013; Wang et al., 2025) all occurred in pediatric ICUs. One (Otag et al., 2005) involved two patients diagnosed within a 20-day period in a Turkish pediatric ICU in 2005. Another (Liu et al., 2013) occurred in the NICU of a Chinese children’s hospital in 2013. The most recent (Wang et al., 2025), also in China, resulted in infections in four neonates. Six patients developed hospital-acquired *K. ohmeri* fungemia within 5 months. Clonality was confirmed by Random Amplification Polymorphic DNA (RAPD). All six were low-birth-weight preterm infants with prolonged broad-spectrum antibiotic exposure; five had PICCs, four required mechanical ventilation. Five were successfully treated with caspofungin and one with fluconazole. In the current outbreak, five NICU patients developed *K. ohmeri* fungemia within 2 months. Microsatellite genotyping confirmed the isolates were clonal, indicating nosocomial cross-transmission. All five patients had risk factors (mechanical ventilation, catheterization). Although no formal institutional investigation was conducted at the time of the outbreak, potential causes—such as ward overcrowding, inadequate environmental disinfection, and suboptimal hand hygiene compliance among healthcare workers (HCWs)—are inferred based on previous research. A 17-month epidemiological investigation of 38 *K. ohmeri* fungemia cases in Indian (Chakrabarti et al., 2014) found 78.9% originated in the NICU; contemporaneously, *K. ohmeri* was isolated from HCW hands in the same NICU, strongly implicating HCW hands as the primary transmission route. This aligns with findings by Lotfinejad et al. (2021). Strict hand hygiene and rigorous environmental disinfection are therefore critical for preventing and controlling NIs in NICUs. Surveillance using methods such as microsatellite genotyping can effectively reduce NI rates (Li et al., 2017) and have been proved a new tool for hospital infection surveillance (Sabino et al., 2015; Luo et al., 2023; Gewecke et al., 2024). Microsatellites, defined as short tandem repeats dispersed throughout the genome, are identified by motif searches. These loci are highly polymorphic and demonstrate excellent inter-laboratory reproducibility compared to traditional methods like RAPD (Zhang et al., 2020; Li et al., 2024). Using microsatellite genotyping, we confirmed a nosocomial infection outbreak that had not been previously detected or managed, demonstrating its value in NI surveillance. Unfortunately, as this outbreak was identified retrospectively, environmental sampling for transmission route tracing was unavailable. Moreover, because the outbreak was recognized and analyzed retrospectively, our findings could not be applied to real-time infection control measures during the actual event. This underscores the need for prospective surveillance systems that enable early detection and prompt intervention in neonatal intensive care units.

CHROMagar Candida chromogenic medium is a rapid and convenient tool assisting in the routine identification of common Candida species based on characteristic colony color changes. However, the color of *K. ohmeri* colonies on this medium changes dynamically over time, transitioning from pink to blue. This phenomenon typically requires at least 2–3 days for pink-blue colonies to develop, and a full week may be needed for complete blue colony formation. If colony colors are assessed at inappropriate times (too early or too late), *K. ohmeri* can be easily confused with other *Candida* species. In this study, all isolates exhibited this color transition, which likely explains their initial misidentification as *C. albicans* (*n* = 6) and *C. dubliniensis* (*n* = 1), both of which typically appear as green colonies on CHROMagar. Such identification confusion has been previously reported; for instance, Indian investigators (Chakrabarti et al., 2014) retrospectively identified 38 *K. ohmeri* isolates among 148 presumed *C. tropicalis* strains. Consequently, clinical microbiology laboratories are advised to use MALDI-TOF MS or gene sequencing for accurate identification of yeast isolates.

Morphological transition in *Yeast*, that is their ability to switch reversibly between yeast and hyphal/pseudohyphae forms, represents a crucial biological characteristic and a well-established pathogenic mechanism. This phenomenon has been extensively studied, particularly in *Candida albicans* (Gow et al., 2011; Beaussart et al., 2012, Villa et al., 2020). The hyphal/pseudohyphae form exhibits enhanced adhesion and tissue invasion capabilities, enabling biofilm formation and resistance against host immunity (Chow et al., 2021). Conversely, the yeast form facilitates dissemination through the bloodstream to distal organs and possesses a greater capacity for immune evasion (Gow et al., 2011). Both forms play significant roles in invasive fungal diseases, especially bloodstream infections caused by yeast. The ability to switch between these two morphological states is a key virulence trait for pathogenic yeasts (Brown and Gow, 1999), mutants locked in either hyphal/pseudohyphae or yeast form demonstrate diminished virulence (Lo et al., 1997). Factors such as elevated temperature (37°C), the presence of serum, contact with endothelial cells, and interaction with other microorganisms within the host environment (Grainha et al., 2020) can induce the morphological transition from the yeast to hyphal/pseudohyphae. In this study, we observed morphological polymorphism in strain *K5* and hypothesized that *K. ohmeri* may possess a similar adaptive mechanism. The strain *K5* was isolated from a 1-month-old neonate admitted to the NICU with fever. The pathogen was isolated 1 week after NICU admission, and only 2 days after the previous case. Therefore, we hypothesized that the pathogen might have been transmitted via air or HCWs’ hands. Subsequently, it converted to the hyphal/pseudohyphae form, expressing adhesins and forming a biofilm on the PICC catheter. Switching back to the yeast form facilitated the detachment of yeast cells, leading to hematogenous dissemination. Peripheral blood samples were collected during this transition period, ultimately yielding colonies exhibiting two distinct morphologies on culture media. Currently, there is no existing research on the morphological transition mechanisms in *K. ohmeri*. Our hypothesis is based on the theoretical framework of morphogenesis dynamics observed in *Candida* bloodstream infections (Cleary et al., 2016; Mech et al., 2014). While the overarching mechanisms are likely similar, differences exist even among various *Candida* species (Staniszewska, 2020). Therefore, specific aspects of morphological transitions in *K. ohmeri*, such as its biofilm-forming capacity, the specific signaling pathways involved, and the molecular targets engaged, require further investigation.

Fluconazole is the most common empiric antifungal agent. Consistent with literature reports (Zhou et al., 2021; Yang et al., 2009; Sundaram et al., 2011; Vivas et al., 2016), our isolates exhibited high fluconazole MICs (4–8 μg/mL), indicating reduced *in vitro* susceptibility. However, two patients were treated with fluconazole solely and recovered, probably due to catheter removal at the same time, which played a decisive role in improving their condition. Several studies (De Barros et al., 2009; Kanno et al., 2017; Haidar et al., 2021; Li et al., 2022) recommend echinocandins as first-line therapy for *K. ohmeri* fungemia due to higher *in vitro* susceptibility and favorable safety profiles compared to azoles. Nevertheless, echinocandin treatment failures have occurred (Distasi et al., 2015; Tashiro et al., 2018). Importantly, echinocandins exhibit poor penetration into sites like the eyes, brain, and cerebrospinal fluid, combination antifungal therapy should be considered for infections involving these sites (Felton et al., 2014). The European Society of Clinical Microbiology and Infectious Diseases (ESCMID) and European Confederation of Medical Mycology (ECMM) joint clinical guidelines recommend amphotericin B as primary therapy for invasive *K. ohmeri* infection, with echinocandins as a second-line option. Arendrup et al. (2014) However, amphotericin B is highly nephrotoxic and requires lower doses in neonates and patients with organ dysfunction. Taj-Aldeen (Taj-Aldeen et al., 2006) reported a case with *in vitro* amphotericin B susceptibility (MIC 0.064 μg/mL) that failed clinically. The patient subsequently responded to liposomal amphotericin B (L-AmB). This underscores that *in vitro* susceptibility does not invariably predict clinical efficacy. Growing evidence (Taj-Aldeen et al., 2006; Sundaram et al., 2011; De Barros et al., 2009) suggests L-AmB may be more effective in neonates. Therefore, considering its safety profile and clinical efficacy, L-AmB may be an alternative option for severe or fluconazole-resistant cases.

In conclusion, *K. ohmeri* is an emerging opportunistic fungal pathogen posing a significant threat to human health. Its environmental presence facilitates nosocomial transmission, particularly within high-risk settings such as NICU. Implementing effective surveillance and stringent prevention measures is critical for controlling nosocomial infections. Accurate pathogen identification and administration of effective antifungal therapy are essential for optimizing patient survival. A key limitation is that although microsatellite genotyping confirmed the nosocomial outbreak, the pathogen source could not be traced due to absence of environmental sampling—including medical equipment surfaces, healthcare workers’ hands, and ward air. Furthermore, the retrospective nature of this investigation prevented the use of these findings for real-time infection control during the outbreak, highlighting the importance of implementing active surveillance in high-risk settings.

## Data Availability

The original contributions presented in this study are included in the article/Supplementary material, further inquiries can be directed to the corresponding authors.
