# Supplementary material for: Outbreak of Kodamaea ohmeri fungemia in neonates: case series and literature review
Source: Front Microbiol. 2026 Apr 7;17:1810513. doi: 10.3389/fmicb.2026.1810513 (PMC13095704; doi:10.3389/fmicb.2026.1810513)
Supplement: Supplementary file 1 [file Table_1.docx]

Supplementary Material

# Supplementary Table

# Supplementary Table 1. Summary of clinical characteristics of *K. ohmeri* fungemia in the medical literature.

| **No.** | | **Year（Ref.)** | **Country** | **Age(Y)** | **Gender** | **Underlying disease** | **risk factors** | **Isolation sources** | **Clinical manifestation** | **Antifungal agents** | **antibiotics** | **MV** | **catheter** | **Other treatment** | **Hospital stays (D)** | **Outcome** |
| --- | --- | --- | --- | --- | --- | --- | --- | --- | --- | --- | --- | --- | --- | --- | --- | --- |
| **Sporadic** | | | | | | | | | | | | | | | |  |
| 1 | 1998 (Bergman et al., 1998) | USA | 48 | F | DM, CAD; renal transplantation | Immuno-compromise | B, C | Fever, severe epigastric discomfort, nausea vomiting | FZ, AMB | VA, CRO | N | CVC, TPN | no | 14 | Died |  |
| 2 | 2000 (Matute et al., 2000) | Nederland | 71 | M | DM, abdominal surgery, endocarditis | Immuno-compromise | B, C, urine, sputum, throat, rectal, spleen | Fever, chills, malaise | AMB | AMC, FLU, CAZ, CRO, RIF, MTZ | Y | Intravenous catheter | Catheter removal | 6 | Died |  |
| 3 | 2003 (Shin et al., 2003) | Korea | 59 | M | Pneumonia | Immuno-compromise | B, skin | Febrile, pain and swelling in the right leg | AMB | FEP, AK, MEM, VA | N | No | No | 68 | Recovered |  |
| 4 | 2004 (Han et al., 2004) | USA | 14 | M | ALL | Catheter implantation | B, C | Tachycardia, fever | FZ | MOX, SXT, FEP | N | CVC | No | 6d | Recovered |  |
| 5 | 2006 (Taj-Aldeen et al., 2006) | USA | Neonate | F | Low weight premature infant, necrotizing enterocoli | Prematurity | B | NM | AMB,  L-AMB | AK, PEN, FEP | Y | CVC | NM | 89 | Recovered |  |
| 6 | 2006 (Ostronoff et al., 2006) | Brazil | 58 | F | CML | Antimicrobial chemoprophylaxis | B, C | Acute epigastric pain, diarrhea, fever, chills | AMB | VA, MEM | N | CVC | CVC removal | 14 | Recovered |  |
| 7 | 2006 (De Barros et al., 2009) | Brazil | 3 | F | Pneumonia, paralytic ileum, parasitization | Exploratory laparotomy | B, C | Acute fever | L-AMB | VA, IPM, GEN | N | CVC | CVC removal | 56 | Recovered |  |
| 8 | 2008(Mahfouz et al., 2008) | Lebanon | 38 | F | Haemochromatosis, AML | Iron overload | B | Abdominal pain | FZ, AMB | IPM | N | Polysite catheter | Catheter removal | NM | Recovered |  |
| 9 | 2009(Yang et al., 2009) | Taiwan | 71 | M | DM, tinea pedis | Cellulitis of the lower extremities | B | Consciousness, fever, bilateral lower leg swelling | FZ, AMB | OXA, IPM-CIP | NM | NM | NM | 20 | Recovered |  |
| 10 | 2009 (Poojary and Sapre, 2009) | India | Neonate | M | Low weight premature infant | Prematurity, very low birth weight, broad spectrum antibiotics | B | Respiratory distress | FZ, AMB | NM | Y | Arterial and venous catheters | Weaned-off ventilator and extubated, Catheter removal | 13 | Died |  |
| 11 | 2010 (Chiu et al., 2010) | Taiwan | 55 | M | Alcoholic hepatitis, peptic ulcer disease | Catheter implantation | B | Fever | FZ, CAS | NM | NM | CVC, TPN | CVC removal | NM | Recovered |  |
| 12 | 2010 (Shaaban et al., 2010) | USA | 34 | M | Asthma, Cardiothoracic surgery | Catheter implantation | B | Febrile, pain at the site of peripheral venous catheter insertion | MF | VA | NM | PICC | PICC removal | NM | Recovered |  |
| 13 | 2010 (Shang et al., 2010) | Taiwan | 71 | M | DM, CAD, Cushing’s syndrome | NM | B | Fever, consciousness, cellulitis | FZ, AMB | OXA | N | N | N | NM | Recovered |  |
| 14 | 2010 (Shang et al., 2010) | Taiwan | 58 | F | Cancer | Surgery, Catheter implantation | B | Fever | FZ | IPM | NM | CVC | NM | 45 | Died |  |
| 15 | 2011 (Al-Sweih et al., 2011) | Kuwait | neonate | F | Bacteremia, low weight premature infant | Bacteremia, broad-spectrum antibiotics used | B | Fever | AMB | AK, MEM, VA | Y | NM | NM | 66 | Recovered |  |
| 16 | 2011 (Sundaram et al., 2011) | India | Neonate | M | Endocarditis | Twins | B | Deeply icteric | AMB | TZP, AK | NM | CVC | Packed cell transfusion | NM | Died |  |
| 17 | 2015 (Distasi et al., 2015) | Italy | 80 | M | MI, COPD, hypertension | NM | B | Not febrile | FZ, AMB | TZP, TGC, colistin | Y | CVC | Tracheotomy | 38 | Died |  |
| 18 | 2016 (Vivas et al., 2016) | Colombia | Neonate | M | NEC | Prematurity | B | Fever, tachycardia | FZ | AMC, VA, MEM | Y | CVC | CVC removal | 36 | Recovered |  |
| 19 | 2017 (Kanno et al., 2017) | Japan | 58 | F | Respiratory failure, pancreatitis | Catheter implantation | B, C | Fever, chill | MF | MEM, TZP | Y | CVC | CVC removal | 33 | Recovered |  |
| 20 | 2018 (Tashiro et al., 2018) | Japan | 60 | M | Severe extended burn | Autograft, Catheter implantation | B, wound | NM | MF, AMB | CAZ, TZP | NM | CVC | NM | 31 | Died |  |
| 21 | 2018 (Diallo et al., 2019) | France | 81 | F | Pneumonia | NM | BALF, B | Fever, vomiting | FZ, CAS, VOR | AMC | N | N | N | NM | Recovered |  |
| 22 | 2021 (Jayaweera et al., 2021) | India | 46 | M | Tricuspid valve myxoma | NM | B | Fever, malaise, tiredness | L-AMB | Beta-lactam, quinolones | N | N | N | 42 | Recovered |  |
| 23 | 2021 (Haidar et al., 2021) | USA | 5 | M | Short gut syndrome | TPN via a CVC | B, C | Productive-cough, rhinorrhea, fever | FZ, MF | VA, FEP | N | CVC | CVC removal | 6 | Recovered |  |
| 24 | 2022 (Li et al., 2022) | China | 37 | M | IFD | Took homemade Chinese herbal medicinal wine | B, Stool | Fever, chills | CAS, VOR | VA, TGC, MEM, LZD, MOX | N | N | N | 51 | Recovered |  |
| 25 | 2022 (Wang et al., 2022) | Taiwan | 52 | M | HIV | Cellulitis, mucocutaneous broken | B, wound | Fever, leg edema, redness, progressive pain | FZ, AMB | NM | NM | CVC | Surgical debridement | NM | Recovered |  |
| 26 | 2022 (Farooq et al., 2024) | Malaysia | Late-30s | M | COVID-19, CAP | NM | B | NM | AND | FEP, MEM, SXT, colistin | NM | NM | NM | 36 | Died |  |
| 27 | 2023 (Sun et al., 2024) | China | 66 | M | Hypertension, DM, PTE, DVT | Poorly controlled long-term hyperglycemia | B, urine | Fever, chills, sweating | FZ | IPM, LEV | NM | NM | NM | 14 | Recovered |  |
| 28 | 2024 (Singh et al., 2024) | India | 22 | M | Suspected cushing | corticosteroid, CVC, broad-spectrum antibiotics | B | Fever | CAS | TZP, TEC, MEM | N | CVC | CVC removal | 27 | Died |  |
| 29 | 2024 (Singh et al., 2024) | India | 81 | F | CKD, hypertension, cholelithiasis | Hemodialysis, long-term TZP usage | B | Fever and pain abdomen | CAS | TZP, MEM, TEC | NM | NM | ERCP | NM | Died |  |
| 30 | 2024 (Singh et al., 2024) | India | 21 | M | Scald | Lost skin | B | Fever | MF | MTZ, VA, CSL, PB | Y | NM | NM | 35 | Died |  |
| 31 | 2024(Guo et al., 2025) | China | 70 | F | DM, hypertension,  Radical gastrectomy | immune dysregulation, mucosal barrier breakdown | B | Fever | VRC | VA, IPM, AK | Y | Closed suction  drains | NM | 48 | Recovered |  |
| **Outbreak** | | | | | | | | | | | | | | | |  |
| 1 | 2005 (Otag et al., 2005) | Turkey | 8M | M | Encephalitis | NM | B | Fever, anorexia, convulsions, consciousness | FZ | MEM, VA | NM | NM | NM | 21 | Died |  |
|  |  |  | 10 | M | ALL | NM | B | NM | AMB | FEP | NM | NM | NM | 21 | Recovered |  |
| 2 | 2013 (Liu et al., 2013) | China | Neonate | M (n=3） | Thrombocytopenia, anemia(n=4)  Sepsis(n=1),  Encephalitis(n=2) | Prematurity | B(n=6)  C(N=2) | Dyspnea, fever, purpura, tachycardia, gastrointestinal symptom | CAS(n=5),  FZ(n=1) | NM | Y(n=4),  N(n-2) | PICC(n=4) | NM | 15-30(n=5),  30(n=1) | Recovered |  |
|  |  |  |  | F (n=3) |  |  |  |  |  |  |  |  |  |  |  |  |
| 3 | 2025 (Wang et al., 2025) | China | Neonate | M(n=3)  F(n=1) | ELBW(n=3),  VLBW(n=1) | Intravenous nutrition,  Endotracheal intubation, PICC | B(n=4)  C(n=3) | Fever | FCA(n=4)  AMB(n=3)  VRC(n=1) | TZP, MEM(n=4)  VA, IPM(n=1) | Y(n=3),  N(n=1) | PICC(N=4) | NM | 71 in average | Recovered (n=3）  Died(n=1) |  |
|  |  |  |  |  |  |  |  |  |  |  | |  | | | |  |

AK, Amikacin; AMB, Amphotericin B; AND, Anidulafungin; ALL, Acute lymphoblastic leukemia; AMC, Amoxicillin-clavulanate; AML, Acute Myelocytic Leukemia; B, Peripheral blood; BALF, Bronchoalveolar lavage fluid; C, Catheter tip; CAD, Coronary artery disease; CAP, Community-acquired pneumonia; CAS: Caspofungin; CAZ, Ceftazidime; CKD, Chronic kidney disease; CML, Chronic myelogenous leukemia; CRO, Ceftriaxone; CSL, Cefoperazone/Sulbactam; CVC, Central venous catheter; D, Days; DM, Diabetes mellitus; DVT, Deep venous thrombosis; ELBW, Extremely low birth weight; ERCP, Endoscopic Retrograde Cholangiopancreatography; F, Female; FEP, Cefepime; FZ: Fluconazole; FLU, Flucloxacillin; GEN, Gentamicin; HIV, Human immunodeficiency virus; IFD, Invasive fungal disease; IPM, Imipenem; IPM-CIL, Imipenem—cilastatin; L-AMB, Liposome amphotericin B; LEV, Levofloxacin; LZD, Linezolid; M, Male; MF: Micafungin; MEM, Meropenem; MI, Myocardial infarction; MOX, Moxifloxacin; MTZ, Metronidazole; MV, mechanical ventilation; NEC, Acute necrotizing enterocolitis; NM, Not mentioned; OXA, Oxacillin; PB, Polymyxin B; PICC, Peripherally Inserted Central Catheter; PTE, Pulmonary thromboembolism; TGC, Tigecycline; TZP: Piperacillin-tazobactam; Ref., Reference; RIF, Rifampicin; SXT, Sulfamethoxazole trimethoprim; TEC, Teicoplanin; TPN, Total parenteral nutrition; VA, Vancomycin; VLBW, Very low birth weight; VOR, Voriconazole; Y, years old;
